# Energy-Efficient Path Planning for Snake Robots Using a Deep Reinforcement Learning-Enhanced A* Algorithm

**DOI:** 10.3390/biomimetics10120826

**Published:** 2025-12-10

**Authors:** Yang Gu, Zelin Wang, Zhong Huang

**Affiliations:** 1 School of Information and Communication Engineering, Hainan University, Haikou 570228, China; guyangl@hainanu.edu.cn (Y.G.); 24120854100005@hainanu.edu.cn (Z.W.); 2 State Key Laboratory of Marine Resources Utilization in South China Sea, Hainan University, Haikou 570228, China

**Keywords:** snake robot, path planning, A* algorithm, deep reinforcement learning, energy consumption, three-dimensional space

## Abstract

Snake-like robots, characterized by their high flexibility and multi-joint structure, exhibit exceptional adaptability to complex terrains such as snowfields, jungles, deserts, and underwater environments. Their ability to navigate narrow spaces and circumvent obstacles makes them ideal for operations in confined or rugged environments. However, efficient motion in such conditions requires not only mechanical flexibility but also effective path planning to ensure safety, energy efficiency, and overall task performance. Most existing path planning algorithms for snake-like robots focus primarily on finding the shortest path between the start and target positions while neglecting the optimization of energy consumption during real operations. To address this limitation, this study proposes an energy-efficient path planning method based on an improved A* algorithm enhanced with deep reinforcement learning: Dueling Double-Deep Q-Network (D3QN). An Energy Consumption Estimation Model (ECEM) is first developed to evaluate the energetic cost of snake robot motion in three-dimensional space. This model is then integrated into a new heuristic function to guide the A* search toward energy-optimal trajectories. Simulation experiments were conducted in a 3D environment to assess the performance of the proposed approach. The results demonstrate that the improved A* algorithm effectively reduces the energy consumption of the snake robot compared with conventional algorithms. Specifically, the proposed method achieves an energy consumption of 68.79 J, which is 3.39%, 27.26%, and 5.91% lower than that of the traditional A* algorithm (71.20 J), the bidirectional A* algorithm (94.61 J), and the weighted improved A* algorithm (73.11 J), respectively. These findings confirm that integrating deep reinforcement learning with an adaptive heuristic function significantly enhances both the energy efficiency and practical applicability of snake robot path planning in complex 3D environments.

## 1. Introduction

In recent years, snake robots have gained a lot of attention for their applications in complex environments due to their unique locomotion and flexibility. Such robots perform well in terrains such as swamps, forests, and rocks and can easily accomplish tasks such as search, rescue, and inspection. However, how to plan an optimal motion trajectory in three-dimensional complex environments is still a challenging topic.

Currently, most research on path planning focuses on solving the “point-to-point” path problem from a fixed starting point to a target point, while there are relatively few studies on path planning for snake robots in complex environments. Various algorithms have been proposed to deal with the path planning problem, but they still have many shortcomings when facing complex three-dimensional environments.

Ge H et al. proposed a 3D path planning algorithm based on energy consumption optimization [1], which reduces the energy consumption of spherical robots in complex terrain to a certain extent; however, due to the different motion models, it is unable to solve the energy consumption problem of the snake robot’s motion. Li B et al. used an improved A* algorithm [2] which, by optimizing the heuristic function, improves the efficiency and accuracy of path planning; however, there are still limitations in the trade-off between energy consumption and path length. Quinones-Ramirez M et al. introduced a deep reinforcement learning technique [3] with the aim of improving the adaptability of path planning through self-learning and experience accumulation; however, their algorithm’s complexity is high, and realizing real-time computation in resource-constrained embedded systems is difficult.

Traditional path planning methods, such as the classic A algorithm, mainly focus on finding the shortest path based on distance. The A algorithm estimates the cost from the current node to the target node using a heuristic function, usually relying on the Euclidean distance or Manhattan distance for estimation in two-dimensional space. However, these methods do not consider energy consumption or real-time adaptability in dynamic environments, which limits their effectiveness in 3D space. The lack of consideration of factors such as terrain undulations, angle changes, and energy efficiency in motion makes the traditional A* algorithm inapplicable in more complex environments. In addition, although the improved bidirectional A* algorithm has an improved path search speed, its performance in complex terrain and 3D environments is no different from that of traditional algorithms. Therefore, in this study, an improved A* algorithm incorporating deep reinforcement learning is proposed for the path planning of snake robots to effectively reduce energy consumption and enhance path planning efficiency.

The improved A* algorithm based on deep reinforcement learning proposed in this paper optimizes the heuristic function by introducing a deep reinforcement learning model, so that the algorithm not only considers the path length but also dynamically evaluates the energy consumption. In order to verify the effectiveness of the proposed algorithm, a large number of simulation experiments were conducted. The results show that, in a complex three-dimensional environment, the improved algorithm can significantly reduce the energy consumption of the snake robot. By reasonably selecting the path, the improved algorithm makes the robot more efficient in energy use, achieves energy saving, and improves the reliability of task completion.

Compared with the traditional A* algorithm, the bidirectional A* algorithm, and other improved A* algorithms, the proposed method has significantly improved energy consumption and path planning quality. Specifically, the improved algorithm can reduce energy consumption under the same experimental environment while showing better results in terms of path planning accuracy and feasibility. This research result provides important technical support for the widespread deployment of snake-like robots in practical applications. The subsequent sections of this paper are organized as follows. Section 2 presents an analysis and modeling of serpentine locomotion. Section 3 introduces the proposed improved A* algorithm for snake robots, which is based on deep reinforcement learning. This is followed by Section 4, which details comparative simulation experiments that evaluate the proposed method against three existing A*-based path planning approaches. Finally, this paper concludes with a summary and future research directions.

## 2. Snake Robot Motion and Torque Analysis

As a kind of bionic robot that mimics the movement of snakes in nature, the snake robot realizes flexible and efficient movement through the coordinated movement of multiple joints. In order to understand its motion mechanism and further optimize the path planning approach, we need to model its motion and derive the relevant physical quantities. The motion of a snake robot can usually be described with a snake curve. In assuming that the joint motion of a snake robot follows a serpentine curve [4], its curvature equation is

(1) ρ=−αbsin(bs),

where α is the magnitude angle in radians (rad), *b* is a constant of proportionality in radians per meter (rad/m), and *s* is the length of the serpentine curve in meters (m).

Equation 1 is integrated over *s* to obtain an expression for the angle along the curve.

(2) θ(s)=αcos(bs).

According to (2), the relative turning angle between each joint of the snake robot can be expressed by the following equation:

(3) φ=θ(s+l)−θ(s−l)=−2αsin(bl)sin(bs),

where *l* is the half-cell length of the snake robot.

In order to more easily describe the motion of each joint, we can rewrite the above equation as a function of time t in the folowing form

(4) $$\varphi_{i}\left(t\right)=A\sin\left[\left(\begin{array}{c} \omega t+\left(\begin{array}{c} i-1 \end{array}\right)\beta \end{array}\right)\right],$$

where *A* = −2αsin(bl) is the amplitude, related to the amplitude angle of the curvature and the length of the joints; ω*t* = bs is the relationship between the time and length of the curve; β = 2bl is the phase difference, related to the proportionality constant of the curve and length of the joints; *i* indexes the number of the joint, ranging from 1 to *n*; *t* is the time, in seconds (s); and *n* is the number of joints in the robot. These equations can effectively describe the motion of the snake robot over time and lay the foundation for subsequent torque and energy analysis.

During the motion of the snake robot, the motion of the head has the greatest influence on the overall path. We simplify the motion of the snake robot to the motion analysis of the head; so, *i* takes the value of 1. We assume that the angular change in the head of the snake robot is expressed as follows:

(5) $$\phi_{h}\left(t\right)=A\sin\left(\begin{array}{c} \omega t+\beta +\sigma \end{array}\right),$$

where *A* is the original amplitude, indicating the maximum magnitude of the angular change; ω is the angular frequency, controlling the speed of angular change, in radians per second (rad/s); and σ is the phase adjustment factor.

In order to solve the angular velocity of the head of the snake robot, we obtain the angular velocity formula through first-order derivation of the above angular variation function:

(6) $$\phi_{h}^{\prime }=A\omega \cos\left(\omega t+\beta +\sigma \right).$$

Furthermore, because energy consumption is related to the motion of the joints under friction and torque, and because angular velocity describes the rate at which the angle of the head of the serpentine robot changes during motion, it directly affects the torque required. During the motion of the snake robot, the joints need to provide a certain amount of torque in order to overcome the inertial forces and friction; so, we assume that the torque τ is directly proportional to $\phi_{h}^{\prime }$.

The amount of available torque is

(7) $$\tau \left(t\right)=k^{*}\phi_{h}^{\prime }\left(t\right)=kA\omega \cos\left(\omega t+\beta +\sigma \right),$$

where *k* is the resistance coefficient, which depends on the actual situation.

## 3. Improved A* Algorithm for Snake Robots Based on Deep Reinforcement Learning

### 3.1. The A* Algorithm and Its Limitations

Path planning methods for robots can be distinguished into two main categories: traditional algorithms and artificial intelligence algorithms. The A* algorithm proposed in our paper falls into the traditional category, as an improvement on the basis of the traditional A* algorithm [5]. The traditional A* algorithm is a classical graph search algorithm which is used to find the optimal path from a start node to a goal node in a graph or network. Unlike a simple breadth-first or depth-first search, the A* algorithm selects the next node to be explored by combining the actual and expected costs of each node. Specifically, the A* algorithm evaluates the priority of a node using two important metrics: actual cost (g-value) and expected cost (h-value). The starting point is assumed to be point 1 with coordinates ($x_{1}$, $y_{1}$), the goal point is point z with coordinates ($x_{z}$, $y_{z}$), and the current point is point n with coordinates ($x_{n}$, $y_{n}$). In addition, the points that make up the path are denoted by point 1 through point *n*.

The actual cost is the actual path cost from the starting node to the current node, g(*n*), can be expressed as follows:

(8) $$g\left(n\right)=\sum_{i=1}^{n-1}d_{i,i+1}.$$

Moreover, the expected path cost from the current node to the goal node, h(*n*), can be expressed as follows:

(9) $$h\left(n\right)=\sqrt{{\left(x_{n}-x_{z}\right)}^{2}+{\left(y_{n}-y_{z}\right)}^{2}}.$$

The sum of these two values is used as the priority of the nodes, such that nodes with lower cost are prioritized in the search process. Thus, these two values are used to build the heuristic function f(*n*) as follows:

(10) $$f(n)=g\left(n\right)+h\left(n\right).$$

The A* algorithm maintains two lists: an open list stores nodes to be explored, while a closed list stores nodes that have already been explored. The steps of the algorithm are as follows: Add the starting node to the open list and initialize its actual and expected costs to 0, then repeat the following steps until the target node is found or the open list is empty. Select the node with the smallest total cost in the open list as the current node, remove the current node from the open list and add it to the closed list, and then process the neighboring nodes of the current node. By continuously selecting the node with the smallest total cost and expanding its neighboring nodes, the A* algorithm eventually finds the optimal path from the start node to the goal node. This algorithm performs well in many cases, but it also has a few drawbacks. First, it is highly dependent on the choice of heuristic function [6]. If the heuristic function is not accurate enough or does not apply to a particular problem, the algorithm may explore unnecessary paths, leading to performance degradation. Second, the A* algorithm may need to store a large amount of node information in some cases, resulting in high memory consumption [7]. Finally, since the A* algorithm is a greedy algorithm, it may fall into a local optimal solution and fail to find a global optimal solution [8]. To overcome these shortcomings, deep reinforcement learning can be introduced to AI algorithms as an effective solution [9]. Deep reinforcement learning improves the performance of path planning by learning the environment’s characteristics and reward mechanism, and it can adapt to the needs of different environments and problems, thus improving the efficiency and accuracy of path planning. On this basis, its application to snake robots can further demonstrate its advantages. The motion and path planning requirements of snake robots in complex environments are extremely high, and traditional methods often struggle to handle these requirements effectively [10]. Deep reinforcement learning can enable snake robots to achieve efficient path planning and motion control in various complex terrains through continuous learning and optimization strategies [11]. In addition, deep reinforcement learning allows the energy consumption factor to be considered in path planning and the energy consumption to be minimized by optimizing the motion path, thus significantly improving the endurance and overall efficiency of the snake robot. In this way, deep reinforcement learning not only improves the accuracy and adaptability of path planning but also effectively reduces the energy consumption of the snake robot in practical applications.

### 3.2. Deep Reinforcement Learning Framework

The traditional deep reinforcement learning framework mainly consists of several core parts: a state space, action space, reward function, and policy network [12]. In this framework, an intelligent body continuously learns and optimizes its behavioral strategies to maximize the accumulated rewards through interaction with the environment. The state space is a description of the set of all possible states of an intelligent body in a given environment. Each state is represented by a vector that contains all important information about the current state of the intelligent body. The action space is the set of all possible actions that an intelligent body can take in any state. Each action leads to a change in the state and triggers a corresponding reward or punishment. The reward function is used to evaluate the performance after each action and give the corresponding reward or punishment, thus guiding the intelligent body to optimize its behavioral strategy.

**Figure 1:**
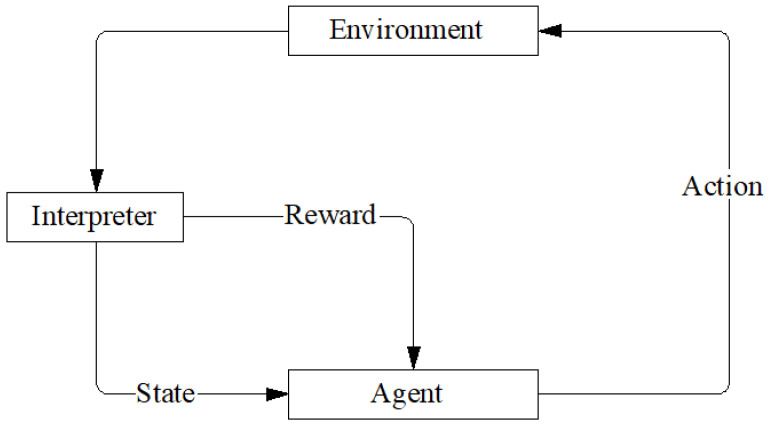
Schematic of deep reinforcement learning.

During the training process, the intelligent body selects actions through a strategy network. The strategy network is usually a deep neural network that outputs the value of each possible action based on the current state. Through continuous trial and error, the policy network is gradually optimized, allowing the intelligent body to select actions that maximize long-term rewards. This process is achieved through reinforcement learning algorithms such as Q-learning or State Action Reward State Action (SARSA) [13] where, by constantly updating the Q-value, the intelligent body is able to gradually learn the optimal strategy. A flowchart is the DRL process is shown in Figure 1.

In this study, in order to reduce the energy consumption of the snake robot in path planning, a D3QN system framework based on deep reinforcement learning is designed and introduced. D3QN, or Double-Deep Q-Network, avoids bias due to the over-estimation in traditional Q-learning by estimating the values of the current and future states separately through two independent neural networks.

In the D3QN system, the state space describes the set of all possible states of the snake robot in a specific environment. Each state includes information such as the coordinates of the robot’s current position, the amount of energy consumed by the turning angle, and the coordinate positions of the surrounding obstacles. This information is fed into the neural network and used to evaluate the value of the current state. In this way, the D3QN is able to capture various possible state changes in complex environments and provide more accurate path planning guidance for the robot [14].

The action space, on the other hand, is the set of all possible actions that the snake robot can take in any state. These actions include basic 3D spatial movement operations such as forward, backward, left turn, right turn, and so on. In each state, the robot selects an action based on the policy network, which leads to a change in the state and triggers a corresponding reward or punishment. Through continuous trial and learning, the D3QN is able to gradually optimize its strategy so that the robot can choose actions that can achieve the goal while minimizing energy consumption.

The reward function plays a key role in the D3QN system; it is used to evaluate the performance of the snake robot after each action and reward or penalize it accordingly. In order to ensure the energy efficiency of path planning, the reward function takes into account not only the movement distance but also the energy consumption. The reward function in this study was designed as follows:

(11) $$r=\left\{\begin{array}{cc} r_{obstacles} & \;if\;pos=obstacles\\ r_{arrive} & \;if\;pos=nogal\\ \left(d_{n}-d_{o}\right)*k_{d}-r_{z}-r_{angle}+r_{addition} & \;\mathrm{otherwise} \end{array}\right.,$$

where $d_{n}$ is the distance between the robot’s newly selected position and the position of the target point, $d_{o}$ is the distance between the robot’s selected position and the position of the target point at the previous moment, and $k_{d}$ is the weight parameter of the distance difference. If the robot chooses a shorter path but the turning angle is too large, resulting in a large amount of energy consumption, the reward function will be lower; on the contrary, a higher reward will be given if the robot chooses a path with a smaller turning angle with lower energy consumption. Meanwhile, when the current position is less than a certain distance from the goal point, adding extra rewards facilitates reaching the goal point quickly. With the extra reward, at every moment, the robot is given a time penalty *t*, which is a value determined in the deep reinforcement learning step to drive the robot to explore shorter paths and reach the goal location faster. When the robot’s position coordinates match an obstacle’s coordinates, the robot receives a negative reward value of −500. When the robot position reaches the goal point, the robot receives a positive reward value of 1000. In this manner, the reward function is able to guide the robot to choose the path with the least change in the angle of movement and the highest energy efficiency.

During training, the D3QN strategy is updated to maximize the cumulative reward by continuously interacting with the environment [15]. Specifically, D3QN uses two Q-networks for alternating updates, one for selecting the best action and the other for estimating the target Q-value. This dual-network structure effectively reduces the bias in Q-value estimation and improves the stability and accuracy of the algorithm. Each time the robot takes an action, the D3QN updates the parameters in the two Q-networks so that it can more accurately predict the value of the future state and adjust its strategy according to the new prediction.

In this manner, the D3QN system framework is able to efficiently plan paths in dynamic and complex environments, significantly reduce the energy consumption of the snake robot, and improve the efficiency and accuracy of overall path planning. With deeper training, D3QN is able to predict the long-term impact of each action with increasing accuracy, enabling the snake robot to excel in a variety of complex environments, not only reaching its goal quickly but also maximizing energy savings. This provides a solid technological foundation for the widespread diffusion of snake robots in practical applications.

### 3.3. Object Movement Optimization on 3D Maps

Based on the traditional A* algorithm, we improved its motion model to make it suitable for path planning in 3D space. The traditional A algorithm is usually used for 2D planar path search, while its limitations are obvious in complex 3D environments. Therefore, a new motion model is introduced to improve the efficiency and flexibility of the algorithm in 3D space.

To adapt to the needs of 3D path planning, we extend the motion directions of the traditional A* algorithm from 8 directions in 2D to 26 directions in 3D. These directions include the basic motion directions along the x, y, and z axes, as well as the diagonal directions between pairs of the three coordinate axes, allowing the algorithm to perform an effective path search in more complex 3D environments. In each direction of motion, we compute not only the base cost considered by the traditional A* algorithm for movement but also the vertical_cost, which is calculated based on the Euclidean distance; the vertical_cost introduces a weighting factor to adjust the movement requirement on the z-axis. The setting of this weighting factor can be adjusted according to specific application scenarios to adapt to the path planning needs in different environments.

With this improvement, our A* algorithm is able to perform a more flexible and efficient path search in 3D space. The improved motion model not only improves the accuracy of path planning [16], but also significantly improves the performance of the algorithm in dealing with complex 3D environments. This improved method provides an effective solution for 3D path planning, which has important theoretical and practical application value.

### 3.4. Optimizing the A* Evaluation Function

In the traditional A* algorithm, the heuristic function plays a crucial role in path planning. It guides the search direction by estimating the cost from the current node to the target node, thus improving the efficiency of the algorithm. However, when dealing with complex environments, traditional heuristic functions may lead to an inefficient search or fall into local optimal solutions due to inaccurate estimation. Therefore, optimizing the heuristic function is one of the important means to improve the performance of the A* algorithm [17]. We introduce a new heuristic function h to the improved A* algorithm. It combines the difference in heuristic values between nodes and the adjustment factor and consists of the sum of the return value of the deep reinforcement learning action of the negatively trained nodes and the heuristic function, which is expressed as

(12) $$h_{1}=-\mathrm{ch}\left[\mathrm{cn}\left(o\right)\right]+h,$$

where ch[cn(*o*)] is the return value of the current node *o* in the trained neural network, and *h* is the normalized value of the heuristics from the current node *o* to the target node *n* goal [18].

Secondly, the principle of optimizing the evaluation function includes the following points. Consideration of the real cost: The o.cost part ensures the influence of the real cost and guarantees that the path planning process takes into account the real cost of each step, as described above.

Adjustment of heuristics difference: By subtracting current_heuristics, we take into account the influence of the current node heuristics at each step of the computation, thus adjusting the search direction, reducing the turning angle, and obtaining the path with minimum energy consumption. Normalization of heuristics: Dividing h by a constant makes the effect of the heuristics on the overall cost smoother and prevents the search from deviating from the optimal path due to too large or too small heuristics. Finally, the evaluative function values for each step of the optimization are obtained by combining the actual cost, the difference in heuristic values, and the normalized heuristic values.

Optimization effect: The optimized evaluative function effectively balances the influence of the actual cost and the heuristic value, which makes the search process more intelligent, prevents it from falling into the local optimal solution, and improves the search efficiency [19]. At the same time, the stability and reliability of the algorithm in complex environments are ensured through the normalization of the heuristic value. In summary, by introducing the optimization heuristic function, the improved A* algorithm can handle the complex path planning task more efficiently [20], which improves the search efficiency and path planning quality. This optimization method provides strong support for further path planning research and applications.

### 3.5. Energy Consumption Judgment Model

In order to realize more efficient path selection in 3D path planning, we introduce a new energy consumption judging model. The traditional A* path planning algorithm mainly focuses on the path length, ignoring the complexity of energy consumption in the actual moving process. In this study, a more refined energy consumption model is proposed by integrating the path length, angle change, and vertical movement. In 3D path planning, energy consumption is mainly composed of the path length, angle change, and vertical movement. Path length consumption is the most basic energy consumption factor in path planning, as more energy is required with a longer path length. Angle change consumption refers to the additional energy required when a change in direction occurs on the path, which is often overlooked and is an important factor affecting the robot’s runtime. Thus, we calculate the energy consumption caused by the angle. Because the calculation of energy consumption is closely related to the power of the motor of the snake robot, according to torque Equation (7), we can obtain the power equation as

(13) $$P\left(t\right)=\tau *\phi^{\prime }\left(t\right)=kA^{2}\omega^{2}\cos^{2}\left(\begin{array}{c} \omega t+\beta +\sigma \end{array}\right).$$

According to the power equation, we can integrate it to deduce the energy formula as

(14) $$energy\text{\_}cost=\frac{k*A^{2}*\omega^{2}*T}{2},$$

where ω is the angular velocity calculated as $\omega =\frac{\theta }{T}$, θ is the angle change per unit time of movement, *T* is the time interval, and *k* and A are constants set according to the drag coefficient of the snake robot in the water and the snake robot’s curve length, respectively. The formula integrates the effects of angle change and distance traveled on energy consumption. The total angle change consumption can be obtained by calculating the angle change in each segment of the path. Vertical movement consumption takes into account the additional energy required to move vertically in 3D space. Typically, vertical movement consumes more energy than horizontal movement; so, additional weights need to be added when calculating the total energy consumption. The final energy consumption formula combines path length consumption, angle change consumption, and vertical movement consumption. The formula is as follows: path_energy = (path length consumption × path length weight) + (vertical movement consumption × path length weight) + (angle change consumption × angle weight), where path length weights and angle weights are used to balance the weights of different energy consumption sources, respectively. By comprehensively considering these factors, we can obtain a path planning result that is more in line with practical application requirements. This last obtained energy consumption value serves as an important basis for judging the goodness of the improved A* algorithm.

## 4. Experimental Simulation

**Figure 2:**
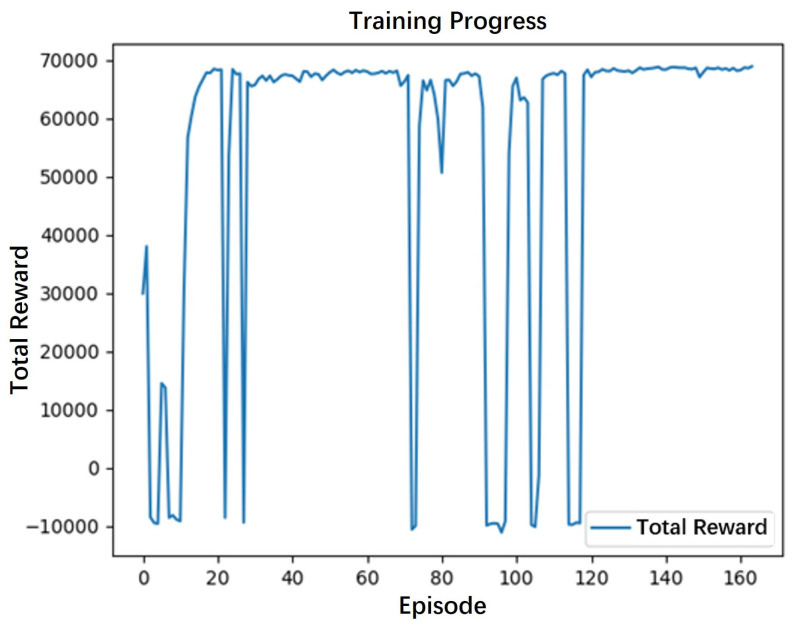
Reward–round diagram.

To verify the effectiveness of the proposed improved A* algorithm for snake robots based on deep reinforcement learning, we conducted comparative experiments against three other methods: the traditional A* algorithm, the bidirectional A* algorithm, and the weighted improved A* algorithm. Initially, the path planning environment (or map) was trained using deep reinforcement learning. To prevent the model from overfitting, an early stopping mechanism was introduced into the training process. This mechanism ensures that the training automatically terminates if the total reward (or average reward) remains unchanged for 50 consecutive epochs (or steps). The training reward curve is illustrated in Figure 2.

Due to the nature of the early stopping mechanism, which only observes the global reward and neglects the convexity of individual rewards, the automated training terminated at the 167th iteration. At this point, the maximum reward was achieved, corresponding to the minimum energy consumption. This convergence signifies that the optimal switching state for the algorithm is reached, after which the A* algorithm is initiated. In the simulation environment, the snake robot’s starting position is marked with a green dot, and the target position is marked with a blue dot.

**Figure 3:**
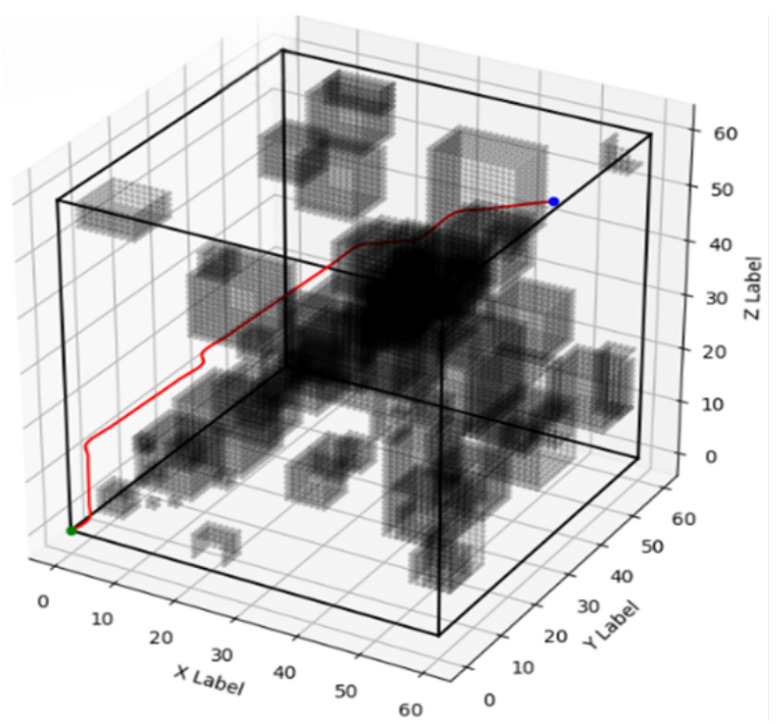
Improved A* path planning diagram.

**Figure 4:**
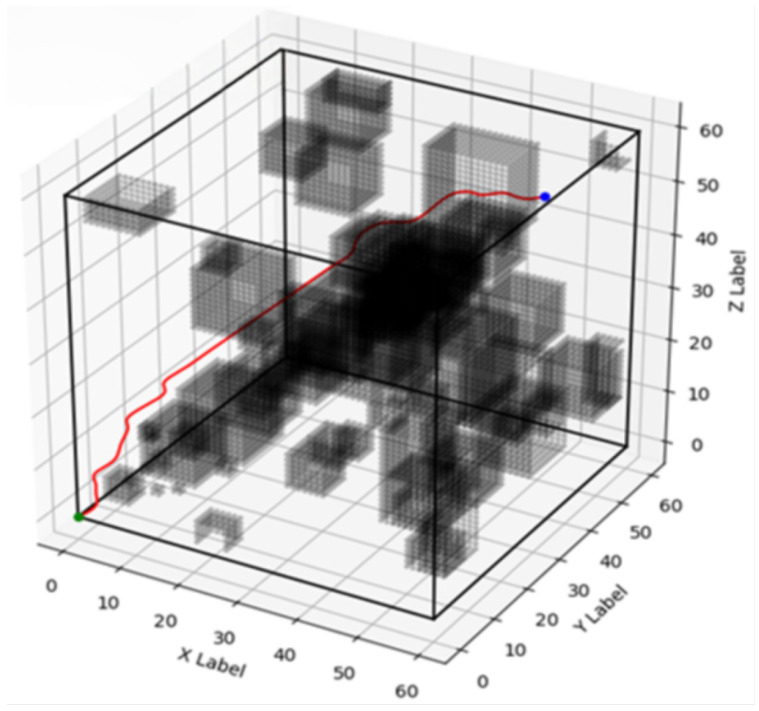
Traditional A* path planning diagram.

**Figure 5:**
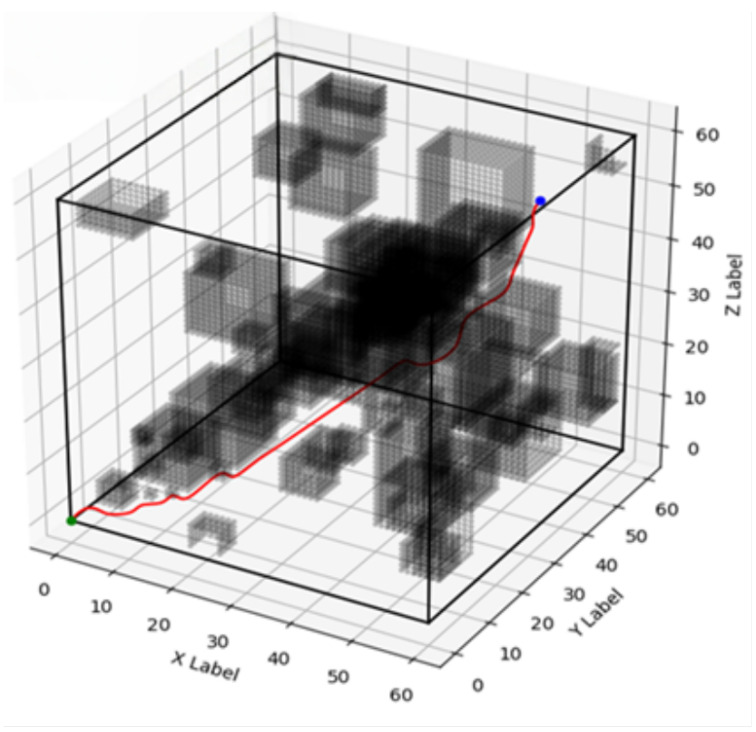
Weighted improved A* path planning diagram.

**Figure 6:**
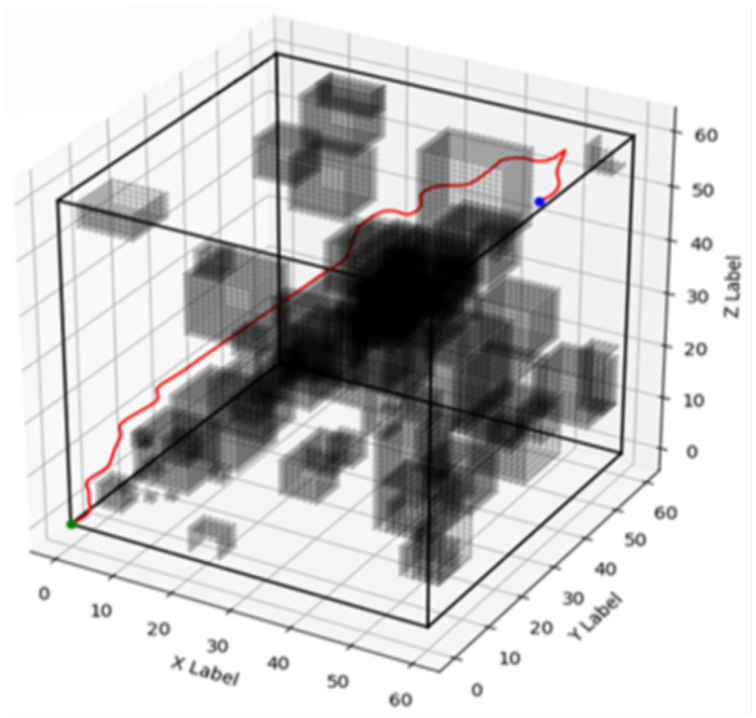
Bidirectional A* path planning diagram.

A series of 200 simulation experiments were conducted for each algorithm—improved A*, traditional A*, weighted improved A*, and bidirectional A*—under identical environmental conditions. The corresponding optimal paths for each algorithm are depicted in Figure 3, Figure 4, Figure 5 and Figure 6, respectively, and a comparative analysis of their performance is summarized in Table 1, with all values representing the averages obtained from the 200 experiments.

From the experimental results, it can be seen that the improved A* algorithm proposed in this paper effectively improves the energy consumption of the paths compared with the traditional A* algorithm, two-way A* algorithm, and weighted improved A* algorithm. Among them, 71.1979 J of energy was consumed by the conventional A* algorithm, 94.6086 J by the bidirectional A* algorithm, and 73.1065 J by the weighted improved A* algorithm. Compared to the above algorithms, the energy consumption of the improved A* algorithm in this paper was 68.7876 J, comprising energy consumption decreased by 3.39%, 27.26%, and 5.91%, respectively, which makes it well suited for devices and systems that need to run for long periods of time or are battery-powered. Furthermore, from the point of view of running time, the improved A* algorithm traversed 16,881 nodes although the running time was 399.9234 s. This number, although seemingly high, actually indicates that it performed a very detailed search in the search space to ensure that the best path was found. Normalized, the improved A algorithm is the most efficient as it is able to perform the most node traversals with lower energy consumption, and the ratio of the number of nodes traversed per unit of time to the amount of energy consumed makes it superior to the other algorithms in terms of overall efficiency, improving the comprehensiveness and accuracy of the path search. The experimental results show that the improved A* algorithm based on deep reinforcement learning proposed in this paper can significantly reduce the energy consumption of the snake robot compared to the traditional A* algorithm. In the experimental scenarios, the improved algorithm exhibited higher path planning efficiency and lower energy consumption. The experiments also show that the introduction of deep reinforcement learning in path planning can effectively optimize the heuristic function and improve the accuracy and reliability of path planning. By comparing the performance of different algorithms in path planning, we can clearly see that the improved A* algorithm based on deep reinforcement learning has obvious advantages in complex 3D environments. The algorithm not only effectively reduces energy consumption but also provides a more reasonable path selection, which enhances the practical application value of snake robots.

## 5. Conclusions

This study proposed an improved A* algorithm based on deep reinforcement learning for optimized path planning for snake robots. By introducing a deep reinforcement learning model and an energy consumption estimation model, we optimize the traditional A* algorithm to obtain a path planning approach with improved efficiency and accuracy. The experimental results show that the improved algorithm can significantly reduce the energy consumption of the snake robot, which provides important technical support for its application in complex 3D environments. The proposed scheme enhances locomotion efficiency primarily for two reasons. Firstly, the reinforcement learning approach enables global optimization of the entire path. Secondly, through incorporation of the kinematic model of the snake robot, the optimized path is better suited for its unique movement patterns.

Therefore, the main contribution of this work is the proposal of a deep reinforcement learning-based method to enhance the A* algorithm for optimal path planning in snake robots. The proposed method reduces both the number of nodes traversed and the energy consumption compared to existing path planning approaches. Despite promising simulation results, this work has certain limitations inherent to its computational nature. The primary limitation is the assumption of an idealized environment, which does not account for real-world challenges such as uneven terrain, sensor noise, or computational constraints on embedded systems. Addressing these factors constitutes a critical direction for our future research. Furthermore, three-dimensional complex environments are highly common in real-world scenarios such as underwater exploration and rescue missions (e.g., earthquake rubble, houses buried by landslides). Therefore, developing an effective path planning methodology for robotic navigation in these settings is of significant practical importance.

## Figures and Tables

**Table 1:** Algorithm comparison table.

Algorithm Name	Running Time	Energy Consumption	Traversing Nodes
Traditional A* algorithm	274.0725	71.1979	3329
Bidirectional A* algorithm	215.2522	94.6086	3150
Weighted A* algorithm	217.6910	73.1065	192
Improved A* algorithm	399.9234	68.7876	16,881

## Data Availability

The original contributions presented in this study are included in this article. Further inquiries can be directed to the corresponding author.
